# Pyoderma Gangrenosum in an Ulcerative Colitis Patient on Vedolizumab

**DOI:** 10.7759/cureus.69219

**Published:** 2024-09-11

**Authors:** Sharon I Choe, Julien Bourgeois, Sreecharita Nidamanuri, Randi Rubenzik

**Affiliations:** 1 Internal Medicine, Creighton University School of Medicine, Phoenix, USA; 2 Dermatology, Southwest Skin Specialists, Phoenix, USA

**Keywords:** biologic, drug reaction, inflammatory bowel disease, pyoderma gangrenosum, ulcer, ulcerative colitis, vedolizumab

## Abstract

Pyoderma gangrenosum is a rare neutrophilic dermatosis that presents as a tender, rapidly progressive ulcer with violaceous, undermined borders. Pathophysiology is multifactorial and has been suggested to involve neutrophil dysfunction, increased T-cell activation, and inflammatory mediator release, often in settings that are known to have autoimmune or genetic diseases. Some medications that modify the immune response have been described to trigger pyoderma gangrenosum. Vedolizumab is a monoclonal antibody used in the management of inflammatory bowel disease that has been shown to be effective in the treatment of pyoderma gangrenosum but, in some cases, has been shown to paradoxically induce pyoderma gangrenosum. Vedolizumab causes gut-selective inhibition of lymphocyte migration, which may lead to the activation of lymphocytes in other organ systems, such as the skin. In this report, we present a case of pyoderma gangrenosum in a patient treated with vedolizumab for ulcerative colitis and explore the possible mechanism behind vedolizumab-induced pyoderma gangrenosum.

## Introduction

Pyoderma gangrenosum (PG) is a rare neutrophilic dermatosis that presents as a tender, rapidly progressive ulcer with violaceous, undermined borders. Classically, PG exhibits pathergy and is triggered by extrinsic injury to the skin in the setting of background immune dysregulation, causing a hyperactive inflammatory response. The disease state involves neutrophil dysfunction, increased T-cell activation, and inflammatory mediator release, often in the setting of known autoimmune disease or genetic disease [1].

Additionally, medications that modify the immune response have been implicated in the development of PG, including granulocyte colony-stimulating factor and small-molecule tyrosine kinase inhibitors [2]. Vedolizumab is a monoclonal antibody used in the management of inflammatory bowel disease (IBD) that has been shown to be effective in the treatment of PG [3]. However, in some cases, it has been shown to paradoxically induce PG [4,5]. In this report, we examine the role of biologic therapy in the onset of PG and present a case of PG in a patient treated with vedolizumab for ulcerative colitis (UC).

## Case presentation

A 30-year-old Caucasian male with a history of UC, rheumatoid arthritis, and hypertension presented to the emergency department with worsening leg and chest ulcers. His UC, which was diagnosed 12 years prior, was controlled with vedolizumab. His vedolizumab regimen was scheduled every eight weeks for the past 20 months at a dosage of 300 mg. He had also tried infliximab in the past but ended up developing antibodies to the medication, which prompted discontinuation.

**Figure 1:**
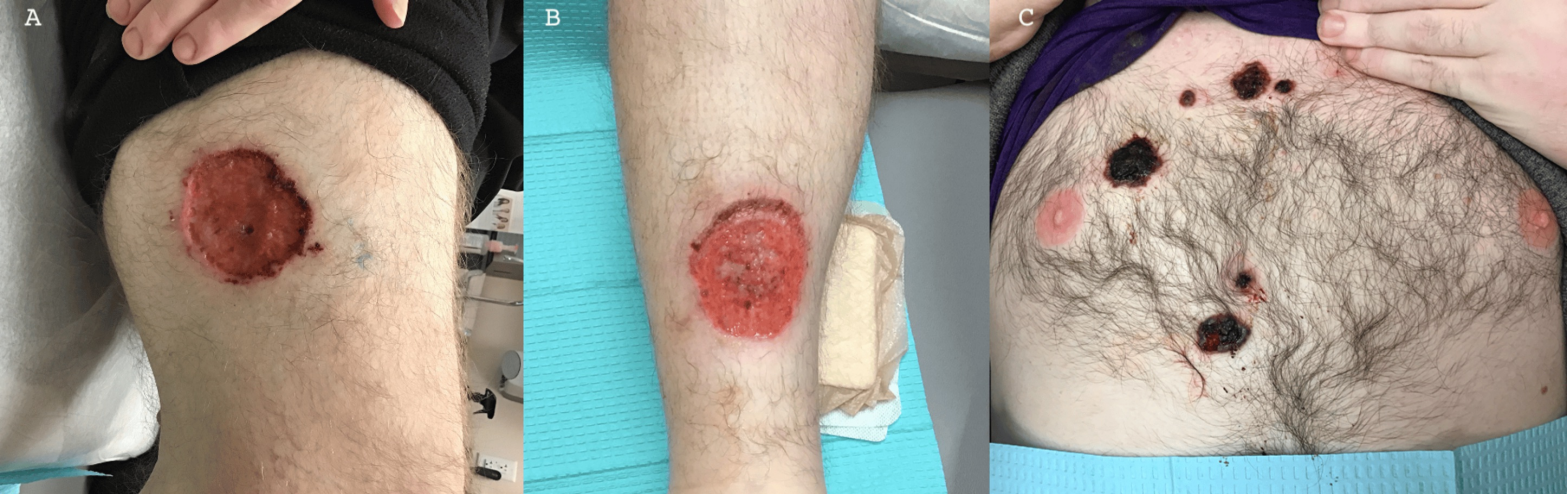
Clinical images of healing ulcers after the initiation of topical steroids, oral doxycycline, and oral prednisone Clinical images of healing ulcers one week after presentation (A) on the lateral thigh and (B) on the anterior lower extremity; (C) persistent hemorrhagic ulcers on the chest one month after presentation

Examination demonstrated an ulcer on the right tibia with well-defined, violaceous edges with a similar lesion on the right thigh (Figures 1A, 1B). Superficial hemorrhagic ulcers with irregular violaceous borders were noted on the chest (Figure 1C). Laboratory investigations revealed low hemoglobin, elevated erythrocyte sedimentation rate, and elevated C-reactive protein. Wound cultures showed heavy growth of *Bacteroides vulgatus* and *Finegoldia magna*. He was started on empiric vancomycin and ceftriaxone and then transitioned to doxycycline.

An initial biopsy of the tibial ulcer revealed an epidermal erosion with a brisk superficial and deep perivascular mixed inflammatory infiltrate. Direct immunofluorescence studies and stains for spirochetes, fungi, and mycobacteria were negative. At this point, differential diagnoses included vasculitis, PG, or an adverse drug eruption. The patient was initiated on topical clobetasol 0.05% ointment and a prednisone taper, starting from 60 mg daily. Doxycycline was also continued for a duration of seven days. His ulcers showed improvement within a week but did not heal.

At his follow-up one month after the initial presentation, a biopsy of a persistent hemorrhagic chest ulcer was performed. This biopsy revealed a striking neutrophilic infiltrate within the dermis, dissolution of the connective tissue matrix beneath the epidermis, an admixture of histiocytes, and granulomatous inflammation. The biopsy also showed secondary vasculitic changes with mural fibrin deposition and extensive red cell extravasation. Some sections showed fibrosis, described as a scar-like process accompanied by neovascularization, reactive perivascular plasmacytosis, and foreign body reaction. Focal areas of robust tissue eosinophils were also noted. Special stains were negative. Vedolizumab-induced PG was favored, and the patient was continued on prednisone. His ulcers improved, but he was hospitalized again five months later for non-healing ulcers.

## Discussion

In a literature search of PubMed using the search terms "pyoderma gangrenosum" and "vedolizumab," we found four other cases of PG in IBD patients on vedolizumab (Table 1). Interestingly, PG ulcers developed while IBD was quiescent in all five cases, including our own. The lack of parallel activity between PG and IBD symptoms in these patients suggests that PG (both de novo PG and PG suspected to be an extraintestinal manifestation of IBD) and gastrointestinal manifestations of IBD may be driven by different mechanisms.

**Table 1:** A literature review of pyoderma gangrenosum in inflammatory bowel disease patients on vedolizumab IBD: inflammatory bowel disease; hx: history; VDZ: vedolizumab; PG: pyoderma gangrenosum; F: female; CD: Crohn's disease; GI: gastrointestinal; IL: intralesional; TAC: triamcinolone acetonide; CsA: cyclosporine; UC: ulcerative colitis, GMA: granulocyte and monocyte adsorptive apheresis; M: male

Study	Age	Sex	Associated Disease	IBD Activity on VDZ	Hx of PG	Duration From Initiation of VDZ to Onset of PG	Management	PG Outcome
Yeh and Tsiaras [6]	32	F	CD	Resolved GI symptoms	Yes	1 month	IL TAC, prednisone, topical cromolyn, topical clobetasol, dapsone, CsA	Improved
Shibuya et al. [7]	50	F	UC	Improved GI symptoms	No	8 months	GMA, prednisolone	Healed
Graziano et al. [8]	16	F	UC	Resolved GI symptoms	No	> 1 year, increased dose frequency	CsA	Healed
Mastronardi et al. [9]	60	F	UC	Improved GI symptoms, Mayo score 0-1	Yes	5 months	GMA	Healed
Our case	30	M	UC	Resolved GI symptoms	No	20 months	Prednisone	Improved

A study comparing IBD patients receiving vedolizumab versus anti-tumor necrosis factor (anti-TNF) therapies found that patients with UC receiving vedolizumab were more likely to develop PG compared to patients treated with anti-TNF agents [5]. Similarly, a retrospective review of 71 patients on vedolizumab revealed that extraintestinal manifestations, most commonly arthralgia, and perianal fistula, occurred in 26.7% of patients with a median onset of 3.75 months between initiation of the drug and extraintestinal manifestation [4]. Of these 71 patients, two patients developed PG [4].

Vedolizumab targets the gut-tropic integrin α4β7, which normally facilitates the migration of T lymphocytes into the gastrointestinal submucosa [4]. This biologic gut-selective mode of action has been demonstrated to be effective in the treatment of UC and Crohn's disease [4]. However, this gut-selective inhibition of lymphocyte migration may lead to the paradoxical activation of lymphocytes in other organ systems [4]. One study found that vedolizumab preferentially replaces pro-inflammatory effector cells, including Th1 and Th17, with regulatory T cells (Tregs) and Th2 cells without globally depleting the intestinal mucosa of lymphocytes [10]. Interestingly, the Treg/Th17 ratio has been shown to be reduced in the skin lesions of PG patients, with a decrease in the number of Tregs and an increase in the number of Th17 cells [11]. Therefore, we hypothesize that vedolizumab-induced PG may be explained by a paradoxical increase in Th17 and a decrease in Treg migration to the skin caused by the selective inhibition of pro-inflammatory effector cell migration to the intestinal mucosa.

Our patient's biopsy was typical for PG in the IBD setting, given the significant histiocytic and granulomatous components. The eosinophilic component was interesting but did not rise to the level of an alternative diagnosis, such as eosinophilic granulomatosis with polyangiitis. The eosinophilia was likely due to co-existing UC [12] or the administration of vedolizumab, which has a significant effect on eosinophil trafficking [13]. Isotopic vedolizumab-induced PG was also considered due to the background of scarring, which may have represented trauma that subsequently triggered PG through pathergy or represented an isotopic response. The patient was continued on vedolizumab to control his UC, and his ulcers failed to heal with systemic steroids. At the time of writing this report, the patient is planning to start a Janus kinase inhibitor, upadacitinib. Previous reports of PG in patients on vedolizumab have reported success with granulocyte and monocyte adsorptive apheresis (GMA) [7,9] and cyclosporine [8]. Both cases treated with GMA were continued on vedolizumab with the improvement of ulcers. Cyclosporine is a non-inferior alternative to corticosteroids that may be a good option to consider in patients with diabetes, obesity, osteoporosis, peptic ulcer disease, or a history of mental illness [14].

## Conclusions

Vedolizumab is a monoclonal antibody used in the management of IBD that has been shown to be effective in both the treatment of PG and paradoxically induce PG. This phenomenon may be caused by selective inhibition of pro-inflammatory effector cell migration to the intestinal mucosa that may subsequently increase pro-inflammatory effector cell migration to the skin. Of the cases reported in the literature thus far, the patients' UC symptoms were well-controlled despite the active skin involvement. Our case suggests that new ulcers in a patient on vedolizumab warrant an index of suspicion for vedolizumab-associated skin manifestations such as PG.
